# Effect of Different Temperatures on the Hydration Kinetics of Urea-Doped Cement Pastes

**DOI:** 10.3390/ma15238343

**Published:** 2022-11-23

**Authors:** Hui Su, Yawei Luan, Qiujuan Ma, Baowen Hu, Shaoxing Liu, Yanjie Bai

**Affiliations:** 1 School of Water Conservancy and Hydroelectric Power, Hebei University of Engineering, Handan 056038, China; 2 Hebei Provincial Key Laboratory of Intelligent Water Resources, Handan 056038, China; 3 Nanjing Hydraulic Research Institute, State Key Laboratory of Hydrology-Water Resources and Hydraulic Engineering, Nanjing 210029, China

**Keywords:** temperature, urea, hydration kinetics, Krstulovic–Dabic model, compressive strength, porosity, hydration mechanism, temperature stress, crack development

## Abstract

Urea can solve the problem of concrete cracking due to temperature stress. However, its effect is affected by temperature. The influencing mechanism of temperature on urea-doped cement pastes is still unclear. This paper explores the effect of different temperatures on the hydration kinetics of urea-doped cement pastes. The isothermal calorimeter (TAM Air) was used to test hydration at three constant temperatures (20 °C, 40 °C, and 60 °C). The effects of the urea admixture and temperature on the hydration process and hydration kinetics parameters were investigated. The hydration mechanism was analyzed, and the changes in macroscopic mechanical compressive strength and porosity were tested. The results show that, as the urea content (UC) increases, the rate of hydration gradually decreases, and the increase in temperature promotes the inhibitory effect of urea. At 60 °C, UC of 8% can be reduced by 23.5% compared with the pure cement (PC) group’s hydration rate. As the temperature increases from 20 °C to 60 °C, the Krstulovic–Dabic model changes from the NG-I-D process to the NG-D process. The effect of urea on the compressive strength of the cement is mainly shown in the early stage, and its effect on later strength is not obvious. In addition, urea will increase its early porosity. The porosity will gradually decrease in the later stage. The results of the study clarify the effect of temperature on urea-doped cement pastes. The optimal content of urea in cement is about 8%, which will provide theoretical guidance for solving the cracking problem of large-volume concrete due to temperature stress.

## 1. Introduction

Cement is a commonly used material in hydraulic buildings [1,2,3,4,5]; however, the phenomenon of cracking concrete due to temperature stress occurs from time to time. The temperature around the environment of a hydraulic building can seriously affect cement hydration [6,7], and reducing the hydration rate of cement to avoid concentrated exotherm is one way to deal with this problem [8]. However, most scholars have mainly focused on reducing the hydration rate of cement under ambient temperature conditions by adding traditional mineral admixtures such as fly ash [9], silica fume [10], and slag [11], etc. Under ambient conditions [12], He et al. [13] mixed fly ash into the cement and tested its 72 h heat of hydration at 20 °C and found that fly ash was able to reduce the total amount of hydration heat from cement hydration for 72 h. The fly ash inhibition is mainly able to bind nucleation. Briki et al. [14] replaced cement with limestone powder and found that limestone powder can promote cement hydration. The mechanism of the influence of traditional mineral admixtures is becoming gradually clear. However, there is less research on the mechanism of the influence of urea [15,16], a common material in daily life, on cement hydration. Urea can reduce the hydration rate in the early stage of curing, thereby reducing temperature stress and avoiding cracks in concrete in the early stage of curing. Park et al. [17] explored the effect of urea on hydration at the normal temperature, noting that the amount of admixture should not exceed 10%. Kim et al. [18] found that, when urea was mixed with concrete, it significantly reduced the ambient temperature around the concrete at the moment of its dissolution.

The Krstulovic–Dabic model of hydration kinetics is one way to understand the hydration process [19,20,21]. Meng et al. [22] used the Krstulovic–Dabic model to study the effect of nano-SiO_2_ on cement hydration and analyzed its mechanism. Changes in cement hydration also cause changes in the microscopic morphology and porosity of the cement pastes [23,24,25,26,27]. Dong et al. [28] observed the morphology of the hydration products through the Electron Microscopy, demonstrating that the nano-silica can make the structure of C-S-H gel more compact. In summary, current scholars studying the hydration effect of urea on cement usually carry out their experiments at 20 °C, and the influence of temperature is rarely taken into account. The effects of urea on cement hydration at different temperatures using the Krstulovic–Dabic model have yet to be further studied.

In this paper, therefore, by testing the hydration rate and the total accumulated hydration heat amount of the cement pastes for 120 h at three constant temperatures (20 °C, 40 °C, and 60 °C), and with urea content (UC) of 0% (PC), 2%, 5%, and 8%, respectively, to replace cement, a comparative analysis was conducted. The Krstulovic–Dabic hydration kinetics model was used to further explore the effects of different urea admixtures on cement hydration under different temperature environments. The effect of urea on the compressive strength of cement paste at different temperatures was analyzed. Finally, according to the pore changes of cement paste, the effect of urea on cement pores under the influence of temperature is further analyzed. This study can provide some theoretical guidance to solve the problem of cement cracking due to temperature stress.

## 2. Materials and Methods

### 2.1. Materials

The cement used was P•O 42.5R ordinary Portland cement produced by Handan Jinyu Taihang Cement Co., Ltd. (Handan, China), and its chemical composition is shown in Table 1. Urea adopts Xilong Science Co., Ltd. (Shantou, China) to produce urea. Its morphology is a white crystalline powder. The physical properties are shown in Table 2. The sand used was the standard sand produced by Xiamen Esio Standard Sand Co., Ltd. (Xiamen, China), and its particle size was 0.08–2.0 mm. The water was deionized water. Table 3 shows the mix proportions of this experiment [29].

### 2.2. Test Method

#### 2.2.1. TAM Air Installation

TAM Air is an isothermal heat conduction calorimeter that operates in the milliwatt range. All calorimetric channels are mounted together to form a calorimeter block that is mounted in a temperature-controlled air thermostat. The TAM Air used in this article is a three-channel version. Each calorimetric channel is constructed in a dual configuration, with a sample on one side and another sample on the other. During the measurement, the sample and reference material are kept in a 100 mL Abe bottle. Measurement data includes hydration rate and total hydration heat release. This data can be continuously recorded in real-time by a computer. In addition, TAM Air thermostats have an operating temperature range of 5 °C to 90 °C and stability of ±0.02 °C.

#### 2.2.2. Test Process

According to the Chinese Standard GB/T17671-2021 test method of cement mortar, sand specimens of 40 mm × 40 mm × 160 mm are required for a suitable strength. We used a WDW-200E microcomputer-controlled electronic universal testing machine to test compressive strength at 3 different temperatures and 4 different dosages for 3, 7, and 28 days. Each working condition casted three groups of specimens, and three groups of specimens were first subjected to a flexural test to obtain six groups of compressive strength test specimens. Then, the average value of the compressive strength of the six groups of specimens for each of these conditions’ compressive strength values was obtained. Obtaining the heat of hydration test included weighing 28 g of cement–urea coagulation mixed into a paper cup and 14 g of deionized water, which was poured it into the paper cup and stirred quickly with a stirring bar for 120 s. Next, we weighed 21 g of cement paste into the TAM Air microcalorimeter special ampoule as the sample group, and used 9.8 g of deionized water into the ampoule as the reference group before placing the ampoule with a lid sealed into the TAM Air microcalorimeter, which was made in the United States. Then, we started the test to assess its hydration rate and the total accumulated hydration heat under three constant temperatures for 120 h. For the microscopic pore test, the test pieces under the completed curing conditions were soaked in anhydrous ethanol, dried, and cracked to select the flatter test pieces from the center of the test pieces. Then, the microscopic morphology and pore changes were monitored using a three-dimensional, ultra-deep field microscope; then, six test pieces were randomly selected for each group of working conditions, and the average value was taken.

### 2.3. Hydration Kinetics Model

The Krstulovic–Dabic model [19,20,21,22] divides the whole process of cement hydration into three basic processes: the nucleation and crystal growth process (NG), phase boundary reaction process (I), and diffusion process (D). The three processes can be carried out simultaneously or sequentially but are mainly determined by the slowest reaction among the three processes [22].

The equations controlling the hydration reaction process for NG, I, and D, respectively, are:

(1) $$\mathrm{NG}:\;{\left[-\ln(1-\alpha )\right]}^{1/n}=K_{1}{}^{\prime }(t-t_{0})$$

(2) $$I:\;[1-{\left(1-\alpha \right)}^{1/3}]=K_{2}{}^{\prime }(t-t_{0})$$

(3) $$D:\;{\left[1-{\left(1-\alpha \right)}^{1/3}\right]}^{2}=K_{3}{}^{\prime }(t-t_{0})$$

where α is the degree of hydration, *t*_0_ indicates the moment when the acceleration period begins, *K*_1_′, *K*_2_′, and *K*_3_′ are the rate constants for the NG, I, and D phases, respectively, *n* is the number of reaction stages, representing the growth of geometric crystals. Differentiating the above Equations (1)–(3), the dynamic differential equation is obtained as:

(4) $$\mathrm{NG}:\;\frac{d\alpha }{dt}=K_{3}{}^{\prime }n(1-\alpha ){\left[-\ln(1-\alpha )\right]}^{(n-1)/n}$$

(5) $$I:\;\frac{d\alpha }{dt}=3K_{2}{}^{\prime }{\left(1-\alpha \right)}^{2/3}$$

(6) $$D:\;\frac{d\alpha }{dt}=\frac{3K_{3}{}^{\prime }{\left(1-\alpha \right)}^{2/3}}{2-2{\left(1-\alpha \right)}^{1/3}}$$

where $\frac{d\alpha }{dt}$ is the reaction rate.

Degree of hydration α in the following equation was used for the calculation:

(7) $$\frac{d\alpha }{dt}=\frac{dQ}{dt}\cdot \frac{1}{Q_{\max}}$$

(8) $$\frac{1}{Q}=\frac{1}{Q_{\max}}+\frac{t_{50}}{Q_{\max}\cdot (t-t_{0})}$$

(9) $$\alpha =\frac{Q}{Q_{\max}}$$

where *Q* is the heat release at the beginning of the acceleration period at moment *t*, J•g^−1^; *Q*_max_ is the total heat release of hydration when the cement paste terminates hydration, J•g^−1^; t_50_ is the time when the total heat release of hydration reaches 50%, h.

## 3. Results and Discussion

### 3.1. Analysis of Hydration Process

#### 3.1.1. Heat of Hydration Analysis

**Figure 1:**
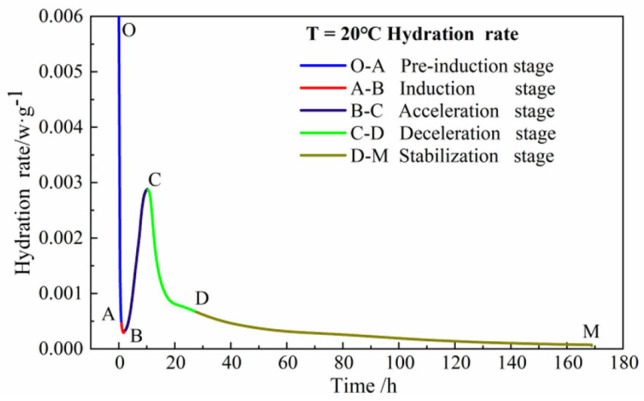
Cement hydration stage division.

According to Figure 1, the hydration process consists of five stages: preinduction, induction, acceleration, deceleration, and stabilization [30].

The O–A section is the preinduction period, mainly C_3_A in cement hydration. The A–B section is the induction section. The B–C section is the acceleration stage. The C–D section is the deceleration phase; at this time, it is the hydration reaction rate with the growth of time and constantly. The last section, D–M, is the stabilization period, and the reaction rate does not change much regarding the growth of time and tends to stabilize, and the structure of this stage becomes denser [31,32].

**Figure 2:**
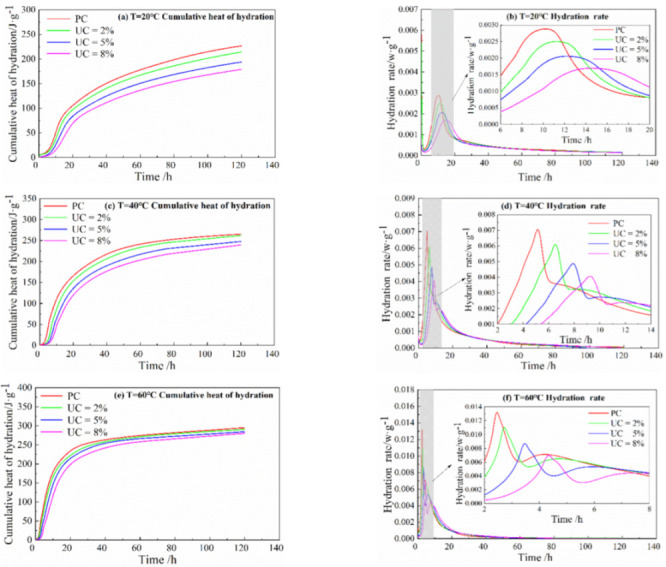
Hydration rate and cumulative heat release graph.

The rate of hydration and cumulative heat release diagrams at different temperatures with different urea blending conditions are shown in Figure 2.

Their values are shown in Table 4.

When the UC is the same, the peak hydration rate gradually increases with the increase in temperature from 20 °C to 60 °C, the time of peak appearance gradually advances, and the total accumulated hydration heat amount increases. UC of 2% at 60 °C increases the hydration rate by 4.4 times compared with 2% of UC at 20 °C. The peak appearance time advances by 7.55 h, and the total accumulated hydration amount increases by 76.432 J·g^−1^, which is because the increase in temperature increases the number of activated molecules in the cement for the reaction, increases the effective collision, promotes the hydration rate of the cement, shortens its induction period, and makes the peak of hydration rate appear earlier.

Under the same temperature condition, an increase in UC reduces the peak rate of the hydration heat, delays peak occurrences, and further reduces this cumulative release heat. Under the condition of 20 °C and UC of 8% compared with PC, the peak hydration rate is reduced by 41%, the peak appearance time is delayed by 4.31 h, and the total cumulative heat release of 120 h is reduced by 47.76 J•g^−1^. While under the condition of 60 °C, with UC of 8% compared with PC, the peak hydration rate is reduced by 66%, and the peak time is delayed by 4.99 h, which shows that urea will inhibit the hydration rate of cement. The reason for this phenomenon is the chemical reaction between urea and water, as shown in Equation (10). This reaction is a heat absorption process, which absorbs the heat around it, inhibiting the hydration of cement. In addition, the addition of urea reduces the content of cement in the cement paste, which reduces the rate of cement hydration to a certain extent.

CO(NH_2_)_2_ + H_2_O = CO_2_ + 2NH_3_ (10)

It can be concluded from the analysis of heat of hydration that the increase in curing temperature will shorten the induction period and make the peak of the hydration rate appear earlier. Urea can inhibit the cement hydration reaction under certain parameters. The inhibition effect is more obvious under the higher curing temperature.

In summary, with an increase in urea content, the peak hydration rate gradually decreases, and the time of peak occurrence is postponed. This indicates that the addition of urea avoids the concentrated heat release of cement. In this way, it can effectively avoid the problem of cracking concrete due to temperature stress in the early stage.

#### 3.1.2. Analysis of Hydration Kinetics

**Figure 3:**
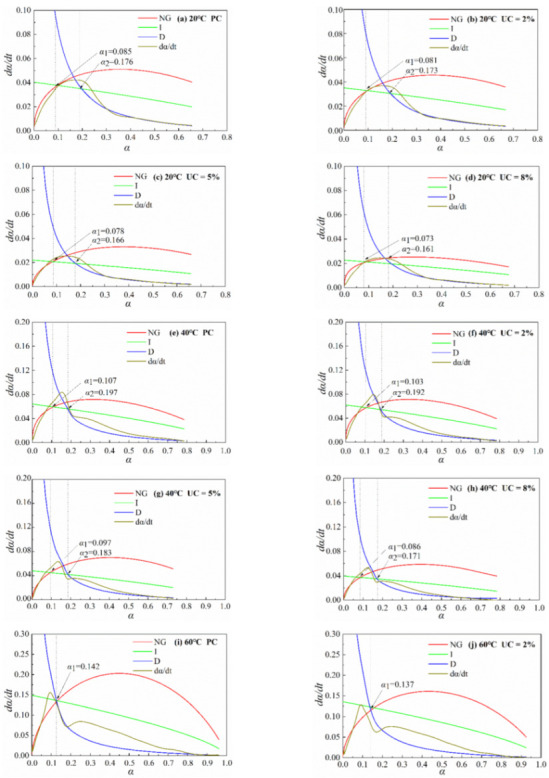

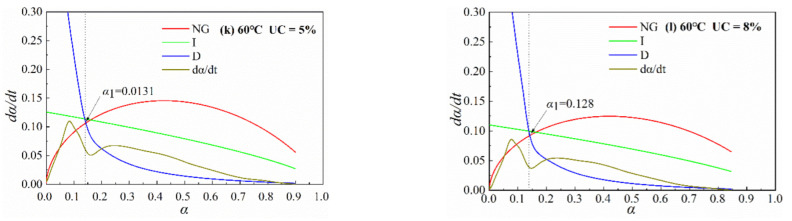
Hydration kinetic model diagram.

Since the preinduction and induction periods last as short as 0–0.5 h, in the actual project pouring, the preinduction and induction periods are often over [30]. Therefore, the hydration kinetics model is simulated from the acceleration period. The hydration kinetics model under different temperatures and different urea admixtures is shown in Figure 3.

The paste under the conditions of 20 °C and 40 °C has experienced three processes of NG, I, and D, indicating that its reaction process is more moderate. Hydration-generated hydration products make a smooth shift in the control mechanism of the hydration kinetics model. In the whole hydration reaction process, in different stages, the main control factors are different. In the early stage of hydration, when the hydration products just began to generate, crystallization nucleation and the crystal growth process (NG) play the main control role. This time is often during the acceleration period. As the generated hydration products gradually increase, the ion migration rate becomes slow. The phase boundary reaction process (I) plays a primary control role at this time in the acceleration and deceleration period, followed by smooth changes in the structure of the paste. Finally, when turned to the diffusion process, (D) plays a primary control role at this time in the deceleration and stabilization period. The whole reaction process rate shows that the phenomenon of the prehydration reaction is intense and affects late reaction stability.

As can be seen from Figure 3, the hydration kinetics model NG, I, and D processes can simulate the actual hydration rate dα/dt curve in a better segment. The process (NG) and the process (D) are better simulated, while the phase boundary reaction process (I) simulation effect has a certain error, which is similar to the conclusion of Zhang et al. [33]. This is because the hydration rate at the peak occurs at this stage, which increases the simulation error. The addition of urea makes *K*_1_′ decrease compared to the PC group. This phenomenon arises mainly because the addition of urea suppresses the cement hydration rate. Urea affects the transition process between the acceleration and deceleration phases, changing the duration of the main control process of the model, but not fundamentally changing the hydration kinetics process.

As the temperature increases, it makes *K*_1_′ increase, so that the NG process begins to shift to the I process at a higher degree of hydration. Even when the temperature increases to 60 °C, the reaction no longer goes through the I process, but directly shifts from the NG process to the D process, which is because the rise in temperature increases the hydration rate of cement, leading to a large number of hydration products in a relatively short period, which accelerates the transformation of the hydration reaction. Thus, it can be seen that the temperature will fundamentally change the hydration kinetics process.

Table 5 shows the kinetics parameters; α_1_ and α_2_ represent the turning point of the NG-to-I process and the turning point of the I-to-D phase, respectively.

As can be seen from Table 5, *K*_1_′ is about 3–5 times as much as *K*_2_′ and about 15–30 times as much as *K*_3_′. The NG process in the three stages is the fastest reaction rate in the whole hydration process, and the D process is the slowest. The reaction rate is relatively fast, and the hydration products grow rapidly. As the reaction proceeds, the hydration process enters the D stage, in which the hydration products make the cement paste pore structure denser. The less permeable C-S-H gel adsorbs on the surface of the unhydrated particles and increases the resistance to further hydrate the unhydrated particles. Therefore, the reaction rate is lowest in the D stage.

The effect of urea’s addition on the K-value was limited: at a temperature of 20 °C, there was a 2% increase in urea admixture, and *K*_1_′, *K*_2_′, and *K*_3_′ values decreased by 12%, 2.5%, and 17.6%, respectively. Moreover, the temperature had a greater effect on *K* values. The temperature raised from 20 °C to 60 °C, and the *K*_1_′, *K*_2_′, and *K*_3_′ values improved by 2.8, 1.75, and 1.2 times, respectively.

After the replacement of cement with urea, the value of n gradually increased, and the values of *K*_1_′, *K*_2_′, and *K*_3_′ all decreased. At the same temperature, the values of *K*_1_′, *K*_2_′, and *K*_3_′ were the highest for the pure cement system, followed by the 2% UC system. The lowest values of *K*_1_′, *K*_2_′, and *K*_3_′ were also found in the 8% UC system. This indicates that different levels of urea have different effects on the growth process of the hydration products and, consequently, on their kinetic parameters. The varying patterns of reaction rates *K*_1_′, *K*_2_′, and *K*_3_′ with urea are consistent with the various patterns of hydration rates with urea, which is consistent with the experimentally measured hydration data, indicating that this model applies to the cement paste in the paper.

The values of *K*_1_′, *K*_2_′, and *K*_3_′ of the cement paste increase as the n value decreases with increasing temperature under the condition of the same urea dosage. The hydration of cement is autocatalytic at the beginning. Many soluble compounds are dissolved from the cement, and the pore solution reaches supersaturation after a few hours. Then, many stable nuclei are formed and start to grow, and temperature increases the rate of dissolution. The supersaturation arrival time is shortened, thus increasing the reaction rate.

### 3.2. Compressive Strength Analysis

**Figure 4:**
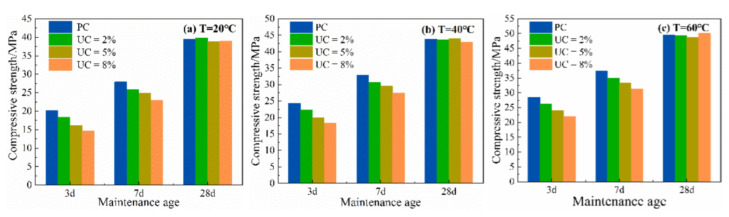
Compressive strength of cement mortar at different temperatures.

The compressive strength of the cement paste at different temperatures and different urea admixtures is shown in Figure 4.

At the same temperature, the compressive strength of the cement paste gradually decreases with an increase in UC at 3 days and 7 days. According to the results of the previous heat of hydration test, it can be assumed that urea inhibits the hydration rate at the early stage of hydration so that the amount of hydration products generated is lower. This is mainly because, with the prolongation of the maintenance period, the amount of Ca(OH)_2_ generated by hydration products gradually increases. The CO_2_ produced by the reaction reacts with the water in the cement paste to produce unstable H_2_CO_3_, as in Equation (11). H_2_CO_3_ reacts with the hydration product Ca(OH)_2_ to generate the stable CaCO_3_ as in Equation (12), which compensates for the large pores generated in the early stage and reduces the porosity, making the cement paste structure denser, and thus increasing the compressive strength in the later stage [17]. Among them, at 20 °C, the compressive strength of the cement paste with 2% UC decreased by 9.5% at 3 days and 8% at 7 days compared to the PC paste, while the compressive strength increased by 1% at after 28 days.

CO_2_ + H_2_O = H_2_CO_3_ (11)

H_2_CO_3_ + Ca(OH)_2_ = CaCO_3_ + 2H_2_O (12)

The temperature increases the hydration rate of cement, so that it produces more hydration products at higher temperatures, leading to the higher mechanical strength of the cement paste. Therefore, the negative effect of incorporating a certain content of urea on its mechanics occurs mainly in the early stage. The mechanical properties will recover to a comparable level with the growth of the period age.

### 3.3. Microscopic Morphology and Pore Analysis

**Figure 5:**
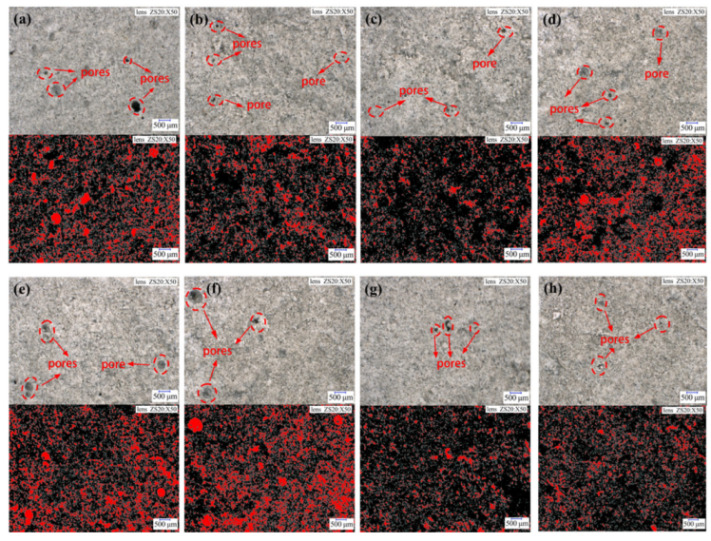
Microscopic morphology and pore distribution of net cement paste under different conditions: (**a**) 20 °C 1d PC; (**b**) 20 °C 2d PC; (**c**) 20 °C 3d PC; (**d**) 20 °C 1d UC = 2%; (**e**) 20 °C 1d UC = 5%; (**f**) 20 °C 1d UC = 8%; (**g**) 40 °C 1d PC; (**h**) 60 °C 1d PC.

Since cement hydration mainly occurred in the first 3 days [34,35,36,37,38,39], super depth-of-field microscopy was used to observe the apparent morphology and pore distribution of the cement net paste specimens under different conditions and analyze the evolution mechanism of their microstructure [40,41,42]. Figure 5 shows the microscopic images of the cement net paste (Zoom in 50×) and the pore distribution images after image segmentation processing (The red color represents the pores).

Table 6 shows the porosity and average pore size under different conditions.

Figure 5 and Table 6 show that, at the same temperature and the same curing age, the lower the urea reference amount, the more uniform and denser the specimen. Moreover, the higher the urea reference amount, the more pore distribution, and mostly the distribution of large pores. The UC increases by 8% and the porosity increases by 7% under the condition of 20 °C at after 1 day. This is because urea itself has strong hygroscopicity and can absorb water molecules around the environment at the early stage of hydration, so that, if there is excess water in the paste, this excess water does not participate in the preliminary hydration reaction, thus resulting in a larger porosity value at the early stage [43,44,45]. In addition, urea inhibits the hydration rate, resulting in the hydration products not being able to fill the pores in time and thus inhibiting the further reduction of the pores.

While the temperature increased from 20 °C to 60 °C and porosity decreased by 16%, the curing period age was extended to 3 days, and the porosity decreased by 14%, which means that the increase in curing time and temperature will promote the generation of hydration products and will compensate for early porosity, so that the pore area is further reduced. In summary, the microscopic pore changes are consistent with the previous thermal and mechanical changes of hydration.

## 4. Conclusions

This paper studied the effects of urea on cement hydration, compressive strength, and microscopic porosity at three constant temperatures. The conclusion is as follows.

1. Urea can reduce the hydration rate, delay the appearance of the peak hydration rate, and avoid the concentration of cement hydration. As the temperature increases, the inhibition effect of urea is better, which can effectively reduce the risk of the cracking of cement due to temperature stress;
2. The hydration kinetics model can effectively simulate the cement hydration process. Urea will make the hydration kinetics model parameters *K*_1_′, *K*_2_′, and *K*_3_′ decrease. The temperature will make it increase. At 60 °C, the Krstulovic–Dabic model reaction no longer goes through process I. Moreover, 60 °C will fundamentally change the kinetics process of hydration;
3. Urea has an effect on the early reduction in compressive strength. However, with the extension of the age of the curing period, its compressive strength will gradually recover to the same level as the compressive strength of PC and has little effect on the later strength; moreover, the higher the temperature, the better its mechanical strength recovery effect;
4. Urea increases the early porosity of cement pastes. The increase in curing time and temperature will gradually reduce the porosity and average pore size.

## Figures and Tables

**Table 1:** Chemical compositions of cement/wt%.

CaO	SiO_2_	Al_2_O_3_	Fe_2_O_3_	SO_3_	MgO	K_2_O	P_2_O_5_	Na_2_O	Loss
64.95	18.31	4.21	2.95	4.22	0.64	0.788	-	-	3.21

**Table 2:** Physical properties of urea.

Molecular Formula	Melting Point/°C	Density g/cm^3^	Water Soluble g/L	Relative Molecular Mass	Crystallization Heat J/g
CH_4_N_2_O	131–135	1.335	1080	60.06	224

**Table 3:** Mix proportion of three groups of 40 mm × 40 mm × 160 mm concrete test pieces.

Components	Cement/g	Water/g	Standard Sand/g	Urea/g
PC	450	225	1350	0
UC = 2%	441	225	1350	9
UC = 5%	427.5	225	1350	22.5
UC = 8%	414	225	1350	36

**Table 4:** Rate of hydration and total cumulative heat release of hydration.

Temperature (°C)	Urea Content	Peak Hydration Rate Time of Occurrence (h)	Peak Hydration Rate (w·g^−1^)	Total Heat Release (J·g^−1^)
20	0%	10.24	0.00287	226.665
	2%	11.29	0.00249	214.154
	5%	12.23	0.00206	193.948
	8%	14.55	0.00169	178.907
40	0%	5.13	0.00704	265.088
	2%	6.52	0.00608	262.071
	5%	7.89	0.00487	247.783
	8%	9.25	0.00407	239.476
60	0%	2.46	0.01319	294.855
	2%	3.74	0.01097	290.586
	5%	5.92	0.00533	284.637
	8%	7.45	0.00442	280.451

**Table 5:** Hydration kinetics parameters.

Temperature (°C)	Urea Content	*n*	*K*_1_′	*K*_2_′	*K*_3_′	*α*_1_	*α*_2_	*α*_2_-*α*_1_	Model Phase
20	0%	1.79	0.0530	0.0120	0.00165	0.085	0.176	0.094	NG-I-D
	2%	1.84	0.0466	0.0117	0.00136	0.081	0.173	0.092	NG-I-D
	5%	1.89	0.0333	0.0073	0.00081	0.078	0.166	0.088	NG-I-D
	8%	1.92	0.0323	0.0075	0.00088	0.073	0.161	0.088	NG-I-D
40	0%	1.61	0.1107	0.0214	0.00214	0.107	0.197	0.090	NG-I-D
	2%	1.66	0.0923	0.0188	0.00228	0.103	0.192	0.890	NG-I-D
	5%	1.72	0.0797	0.0150	0.00179	0.097	0.183	0.086	NG-I-D
	8%	1.85	0.0682	0.0131	0.00130	0.086	0.171	0.085	NG-I-D
60	0%	1.46	0.2022	0.0330	0.00315	0.142	-	-	NG-D
	2%	1.52	0.1691	0.0323	0.00322	0.137	-	-	NG-D
	5%	1.63	0.1564	0.0279	0.00289	0.131	-	-	NG-D
	8%	1.69	0.1135	0.0229	0.00224	0.128	-	-	NG-D

**Table 6:** Porosity and average pore size of net cement paste under different conditions.

Temperature (°C)	Maintenance Period of Age (d)	Urea Content	Porosity	Average Pore Size (μm)
20	1	0%	36%	28.32
	2	0%	28%	27.74
	3	0%	21%	26.21
	1	2%	38%	29.33
	1	5%	40%	30.06
	1	8%	43%	31.45
40	1	0%	25%	27.01
60	1	0%	20%	25.33

## Data Availability

The data presented in this study are available on request the corresponding author.

## References

[1] Wei B., Liu B., Yuan D., Mao Y., Yao S. (2021). Spatiotemporal hybrid model for concrete arch dam deformation monitoring considering chaotic effect of residualseries. Eng. Struct..

[2] Pirooznia A., Moradloo A.J. (2020). Investigation of size effect and smeared crack models in ordinary and dam concrete fracture tests. Eng. Fract. Mech..

[3] Yang D., Gu C., Zhu Y., Dai B., Li B. (2020). A concrete dam deformation prediction method based on LSTM with attention mechanism. IEEE Access.

[4] Singh V.P., Kumar M., Srivastava R.S., Vaish R. (2021). Thermoelectric energy harvesting using cement-based composites: A review. Mater. Today Energy.

[5] Kong X.J., Zhang Y., Zou D.G., Qu Y.Q., Yu X. (2017). Seismic cracking analyses of two types of face slab for concrete-faced rockfill dams. Sci. China Technol. Sci..

[6] Qiu J., Li Y., Xu Y., Wu A., Macdonald D.D. (2020). Effect of Temperature on Corrosion of Carbon Steel in Simulated Concrete Pore Solution under Anoxic Conditions. Corros. Sci..

[7] Valipour M., Khayat K.H. (2020). Robustness of Ultra-High-Performance concrete to changes in material temperature. ACI Mater. J..

[8] Qin C., Gong J., Xie G. (2021). Modeling hydration kinetics of the Portland-Cement-Based cementitious systems with mortar blends by Non-Assumptive projection pursuit regression. Thermochim. Acta.

[9] He J., Long G., Ma K., Xie Y., Cheng Z. (2021). Improvement of the hydration of a fly ash–cement system by the synergic action of triethanolamine and C-S-H Seeding. ACS Sustain. Chem. Eng..

[10] Yang M., Paudel S.R., Asa E. (2020). Comparison of pore structure in alkali activated fly ash geopolymer and ordinary concrete due to alkali-silica reaction using micro-computed tomography. Constr. Build. Mater..

[11] He Y., Liu. S., Hooton R.D., Zhang X., He S. (2022). Effects of TEA on rheological property and hydration performance of lithium slag-cement composite binder. Constr. Build. Mater..

[12] Liu S., Wang L., Gao Y., Yu B., Tang W. (2015). Influence of fineness on hydration kinetics of supersulfated cement. Thermochim. Acta.

[13] He J., Long G., Ma K., Xie Y. (2021). Influence of fly ash or slag on nucleation and growth of early hydration of cement. Thermochim. Acta.

[14] Briki Y., Maciej Z., Haha M.B., Scrivener K.L. (2021). Impact of limestone fineness on cement hydration at early age. Cem. Concr. Res..

[15] Gong C., Zhou X., Dai W., Liu Y., Lu L., Cheng X. (2018). Effects of carbamide on fluidity and setting time of sulphoaluminate cement and properties of planting concrete from sulphoaluminate cement. Constr. Build. Mater..

[16] Kim H.Y. (2021). On the Feasibility of Using Industrial Urea to Mitigate Thermal and Shrinkage Cracking in Concrete. Appl. Sci..

[17] Park J.K., Sang H.H., Hong K.N., Yong I.C., Chai Y. (2016). Influence on Compressive Strength and Drying Shrinkage of Concrete with Urea-Water Soluble Sulfur Admixture. J. Korean Soc. Saf..

[18] Kim H.Y. (2017). Urea additives for reduction of hydration heat in cement composites. Constr. Build. Mater..

[19] Miao M., Liu Q., Zhou J., Feng J. (2019). Early hydration kinetics of expansive cementitious binders. Materials.

[20] Li Z., Gao X., Lu D., Dong J. (2022). Early hydration properties and reaction kinetics of multi-composite cement pastes with supplementary cementitious materials (SCMs). Thermochim. Acta.

[21] Liu L., Yang P., Qi C., Zhang B., Guo L., Song K.I. (2019). An experimental study on the early-age hydration kinetics of cemented paste backfill. Constr. Build. Mater..

[22] Meng T., Hong Y., Wei H., Xu Q. (2019). Effect of Nano-SiO2 with Different Particle Size on the Hydration Kinetics of Cement. Thermochim. Acta.

[23] Qin J., Qin Q., Li X., Xue J., Wang R., Zhang Q., Wang P., Guo Z., Gong Y. (2021). Urea supply control in microbial carbonate precipitation to effectively fill pores of concrete. Constr. Build. Mater..

[24] Zhang X., Yang Z.J., Huang Y.J., Wang Z.Y., Chen X.W. (2021). Micro CT Image-based simulations of concrete under high strain rate impact using a Continuum-Discrete coupled model. Int. J. Impact Eng..

[25] Trofimov A., Mishurova T., Lanzoni L., Radi E., Bruno G., Sevostianov I. (2018). Microstructural analysis and mechanical properties of concrete reinforced with polymer short fibers. Int. J. Eng. Sci..

[26] Liu S., Diederik J. (2017). Coupled reactive transport model study of pore size effects on solubility during cement-bicarbonate water interaction. Chem. Geol..

[27] Wang L.G., Ju S.Y., Chu H.Y., Liu Z.Y., Yang Z.Q., Wang F.J., Jing J.Y. (2020). Hydration process and microstructure evolution of low exothermic concrete produced with urea. Constr. Build. Mater..

[28] Dong P., Allahverdi A., Andrei C., Basim N. (2020). Liquid cell transmission electron microscopy reveals the role of Nano-silica in cement hydration reactions. Microsc. Microanal..

[29] (2021). Test Method of Cement Mortar Strength (ISO Method).

[30] Ridi F., Fratini E., Luciani P., Winnefeid F., Baglioni P. (2011). Hydration kinetics of tricalcium silicate by calorimetric methods. J. Colloid Interface Sci..

[31] Liu K., Cheng X., Gao X., Zhang X., Zhuang J. (2019). Effect of the hydration rate and microstructure of Portland cement slurry on hydrostatic pressure transfer. Powder Technol..

[32] Ding W., Xu W., Dong Z., Liu Y., Shiotani T. (2021). Influence of hydration capacity for cement matrix on the piezoelectric properties and microstructure of cement-based piezoelectric ceramic composites. Mater. Charact..

[33] Zhang H., Yang Z., Su Y. (2019). Hydration kinetics of cement-quicklime system at different temperatures. Thermochim. Acta.

[34] Kopec M., Ranachowski P., Ranachowski Z., Dbowski T., Schabowicz K. (2020). Mechanical and Non-Destructive testing of plasterboards subjected to a hydration process. Materials.

[35] Stroh J., Schlegel M.C., Schmidt W., Nguyen T.Y., Meng B., Emmerling F. (2016). Time-resolved in situ investigation of Portland cement hydration influenced by chemical admixtures. Constr. Build. Mater..

[36] Lin C., Wei W., Hu Y.H. (2016). Catalytic behavior of graphene oxide for cement hydration process. J. Phys. Chem. Solids.

[37] Tan L., Xiao J., Zhu C. (2016). Hydration process modeling of ITZ between new and old cement paste. Constr. Build. Mater..

[38] Choobbasti A.J., Kutanaei S.S. (2017). Microstructure characteristics of cement-stabilized sandy soil using nanosilica. J. Rock Mech. Geotech. Eng..

[39] Asgari H., Ramezanianpour A., Butt H.J. (2016). Effect of water and nano-silica solution on the early stages cement hydration. Constr. Build. Mater..

[40] Kupwade-Patil K., Al-Aibani A.F., Abdulsalam M.F., Mao C., Bumajdad A., Palkovic S.D., Buyukozturk O. (2016). Microstructure of cement paste with natural pozzolanic volcanic ash and Portland cement at different stages of curing. Constr. Build. Mater..

[41] Pu L., Unluer C. (2016). Investigation of carbonation depth and its influence on the performance and microstructure of MgO cement and PC mixes. Constr. Build. Mater..

[42] Pang B., Zhang Y., Liu G. (2018). Study on the effect of waterborne epoxy resins on the performance and microstructure of cement paste. Constr. Build. Mater..

[43] Jiang J., Lu Z., Niu Y., Li J., Zhang Y. (2016). Investigation of the properties of high-porosity cement foams based on ternary portland cement–metakaolin–silica fume blends. Constr. Build. Mater..

[44] Gao Z., Wang L., Yang H. (2021). Investigation of the residual mechanical and porosity properties of cement mortar under axial stress during heating. Materials.

[45] Baleani M., Fognani R., Toni A. (2016). The influence of stem insertion rate on the porosity of the cement mantle of hip joint replacements. Proc. Inst. Mech. Eng. H—J. Eng. Med..

